# Pediatric dental treatment under general anesthesia: a retrospective analysis of clinical indications and a survey of pediatric dentist referral practices

**DOI:** 10.3389/fdmed.2026.1789221

**Published:** 2026-03-11

**Authors:** Avia Fux-Noy, Maya Raz, Karam Masrawa, Aviv Shmueli, Elinor Halperson, Moti Moskovitz

**Affiliations:** 1 Faculty of Dental Medicine, Hebrew University of Jerusalem, Jerusalem, Israel; 2 Department of Pediatric Dentistry, Hadassah Medical Center, Jerusalem, Israel

**Keywords:** dental treatment, general anesthesia, indications, pediatric, practice, referral

## Abstract

**Introduction:**

The utilization of general anesthesia for pediatric dental treatments has increased. This study aimed to investigate the reasons for dental general anesthesia use in a pediatric dentistry department and to survey pediatric dentists' perspectives on dental general anesthesia indications.

**Methods:**

A retrospective cohort study analyzed electronic medical records of patients undergoing dental general anesthesia. Additionally, a questionnaire was distributed to pediatric dentists at professional conferences, collecting responses to seven questions regarding dental general anesthesia indications.

**Results:**

The cohort consisted of 245 dental records. The primary reasons for dental general anesthesia were extensive treatment needs (56%), young age (41%), parental preference (39%), lack of cooperation (27%), and medical comorbidities (23%). Parental preference correlated with the child's behavior during examination (*p* = 0.030). Younger children (<5 years) more frequently underwent dental general anesthesia due to extensive caries, while older children (>5 years) were more likely referred due to behavioral issues and medical comorbidities. Of the 110 dentists who completed the survey, those performing dental general anesthesia were more likely to require a sedation visit before referral (*p* = 0.003) and less likely to consider parental preference alone as an indication (*p* = 0.010) compared to those not performing dental general anesthesia. Dentists performing moderate sedation were less likely to consider multiple visits (*p* = 0.042), multiple extractions (*p* = 0.028), multiple crowns (*p* = 0.047), or parental preference alone (*p* = 0.021) as indications for dental general anesthesia compared to those who did not perform moderate sedation.

**Discussion:**

Partnership with parents and taking their preferences into account are essential in decision-making regarding dental general anesthesia.

## 1 Introduction

The utilization of general anesthesia (GA) for pediatric dental treatments has increased in the last decade (1–5). Diverse factors necessitate dental general anesthesia (DGA), including extensive caries, medical comorbidities, young age requiring complex treatment, prior negative dental experiences, and uncooperative behavior due to anxiety (3, 6–13). According to Rogers et al. (12), the increased use of DGA is attributed to a stronger emphasis on ‘child-centered care,’ as well as perceived low parental competence, parental guilt, and parental convenience. Parents may prefer to avoid any potentially stressful situation and opt for GA, in comparison to prolonged and numerous treatment sessions.

DGA offers several advantages, including the ability to treat uncooperative children without causing pain or awareness, and the completion of extensive treatment in a single session. However, DGA carries a higher risk of complications compared to moderate sedation and local anesthesia, has potential side effects from anesthetic agents, and requires special equipment and a skilled team capable of managing potential complications (6).

The objectives of this study were to determine the factors influencing the use of DGA within the Department of Pediatric Dentistry at Hadassah Medical Center, Jerusalem, and to survey Israeli pediatric dentists regarding their criteria for DGA referrals.

## 2 Materials and methods

### 2.1 Study design and group

This study utilized a dual-method approach, presenting both a retrospective cohort analysis and a survey. The retrospective cohort study examined electronic medical records of patients who underwent DGA in the Department of Pediatric Dentistry at Hadassah Medical Center between 2021 and 2022. The department has the capacity to provide DGA for four children per week. The cohort was limited to two years to capture current data and identify contemporary trends regarding DGA. Referrals for DGA within the department were made by any of the 14 pediatric dentistry specialists, who could choose from the following reasons on an internal departmental form: extensive treatment needs, age, parental preference, lack of cooperation, medical comorbidities, and distance from the hospital. Multiple selections were possible. Criteria for inclusion were patients undergoing DGA with documented reasons for anesthesia noted in their medical records. Data extracted included: gender, age at referral, number of decayed/treated teeth, patient address (for distance calculation), cooperation during examination [using the Frankl behavior scale (8)], dental history, and documented reasons for DGA. The number of decayed/treated teeth was defined by the number of teeth required restorative procedures or extractions. It did not include enamel demineralization or non-cavitated lesions managed with fluoride and other preventive measures.

Concurrently, a questionnaire, adapted from Maccormac and Kinirons (14), was distributed to 122 pediatric dentists attending the Israeli Association of Dentistry for Children professional conference. The association includes approximately 150 members, representing most of pediatric dentists in the country (in Israel, there are 220 licensed pediatric dentistry specialists, although some are retired or no longer practice in the country). The questionnaire consisted of two parts: demographic data and seven questions regarding DGA indications. Inclusion criteria for the survey were Hebrew-speaking and reading pediatric dentists (specialists or general practitioners). Exclusion criteria were incomplete responses to the second section of the questionnaire.

### 2.2 Ethics

This study was conducted in accordance with the principles outlined in the Declaration of Helsinki. Approval was granted by the ethical committee on research involving human subjects (0587-21-HMO and 0400-20-HMO).

### 2.3 Statistical analysis

Data analysis was performed using SPSS software, version 28.0. All statistical tests were analyzed at a significance level of *p*-value < 0.05. For the description of the sample according to demographic variables, frequency distributions and percentages were used. Measures of central tendency and dispersion (standard deviation, minimum, and maximum values) were used to analyze the descriptive statistics of the research variables. For inferential statistics, chi-square tests for independence were conducted to examine the relationship between two categorical variables. In addition, independent samples t-tests were conducted to compare independent samples, segmented by parental preference (yes/no). Fisher test, chi-square test, and multivariate analysis were conducted to analyze categorical variables of the questionnaire.

A *post-hoc* power analysis was performed to evaluate the statistical strength of both study components. For the retrospective clinical arm (*n* = 245), the comparison of categorical variables using Chi-square and Fisher's exact tests (with *α* = 0.05) yielded a statistical power (1-*β*) of 0.98 to detect a medium effect size (Cohen's w = 0.25) and >0.99 for large effect sizes. For the questionnaire survey (*n* = 110), the analysis indicated a statistical power of 0.88 for medium effect sizes (w = 0.30) and >0.99 for large effect sizes. These values exceed the standard 0.80 power threshold, confirming that both sample sizes were sufficient to identify clinically significant associations and minimize the risk of Type II errors.

## 3 Results

Of the 322 children who underwent DGA between 2021 and 2022 and had available medical records, 245 met the inclusion criteria. Seventy-seven were excluded: one patient was treated in a different department, three records were duplicates, and 73 lacked documented reasons for DGA on the internal form. Demographic characteristics are summarized in Table 1.

**Table 1:** Distribution of sociodemographic variables in the sample (*N* = 245).

Variable	Values	Freq. (%)	Mean (SD)	Range
Gender	Male	146 (60)		
	Female	99 (40)		
Medical condition [*n* = 243]	Healthy	159 (65)		
	Developmental behavioral disorder	26 (11)		
	Systemic disease	58 (24)		
Dental History [*n* = 242]	Only examination, no treatment	131 (54)		
	Treated with inhaled/moderate sedation in a different clinic	4 (1.65)		
	Treated with inhaled/moderate sedation in the department	107 (44)		
Residence distance from hospital(km) [*n* = 126]			20.99 (14.85)	5–115
Age at the time of referral for anesthesia (years)			4 (1.98)	2–14
Frankl behavior scale at examination [*n* = 160]			1.8 (1.04)	1–4
Number of decayed teeth			11.44 (3.81)	4–20

Extensive treatment needs were the primary reason for DGA (56%), followed by age (41%), parental preference (39%), lack of cooperation (27%), medical comorbidities (23%), and distance from the hospital (2%) (Table 2).

**Table 2:** Reasons for pediatric DGA.

Variable	Frequency	%
Number of teeth requiring treatment	138	56%
Age	101	41%
Parental preference	95	39%
Behavior and cooperation	67	27%
Health status	57	23%
Residential distance from the hospital	5	2%

Parental preference for DGA was not significantly correlated with the child's dental history [*χ*^2^(1,1) = 3.486, *p* = 0.062, Cramer's V = 0.119]. A significant correlation was observed between parental preference for DGA and the child's behavior and cooperation during the examination [t(160) = −1.899, *p* = 0.030]. Specifically, children whose parents preferred DGA demonstrated higher Frankl behavior scale scores (M = 2.15, SD = 1.17) compared to those where parental preference was not a documented reason (M = 1.73, SD = 1.01). No other variables demonstrated a significant correlation with parental preference for DGA (Table 3).

**Table 3:** Correlations between research variables and parental preference for DGA.

Variable	Documented reason for DGA: parental preference						
	No		Yes		Difference test		
	*M*	*SD*	*M*	*SD*	*T*	*df*	*p*
Age (years)	4.25	2.03	4.19	1.73	196	241	0.422
Residential distance from the hospital (Km)	21.69	15.8	17.04	7.14	1.261	124	0.105
Number of teeth requiring treatment	11.51	3.79	11.08	3.98	665	240	0.253
Cooperation and behavior (Frankl)	1.73	1.01	2.15	1.17	−1.899	160	0.030

A significant association was found between medical comorbidities as a documented reason for DGA and both age (*p* = 0.004) and parental preference (*p* < 0.001). Specifically, younger age and parental preference were more frequently cited as reasons for DGA in healthy children compared to those with medical comorbidities (Table 4).

**Table 4:** Correlations between the child's medical status and DGA reasons (*N* = 245).

Documented reason for DGA		Health status of the child		Total *n* (%)	*p*.
		Medically compromised *n* (%)	Healthy *n* (%)		
Age	No	61 (71)	83 (52)	144 (59)	0.004
	Yes	25 (29)	76 (48)	101 (41)	
Cooperation and behavior	No	63 (73)	115 (72)	178 (73)	0.876
	Yes	23 (27)	44 (28)	67 (27)	
Number of teeth requiring treatment	No	44 (51)	63 (40)	107 (44)	0.082
	Yes	42 (49)	96 (60)	138 (56)	
Residential distance from the hospital	No	84 (98)	156 (98)	240 (98)	0.817
	Yes	2 (2)	3 (2)	5 (2)	
Parental preference	No	67 (78)	83 (52)	150 (61)	<0.001
	Yes	19 (22)	76 (48)	95 (39)	

To analyze age-related differences, the patient cohort was divided into two groups: Those who were under 5 years old and those who were over 5 years old. Significant associations were observed between age as a documented reason for DGA and cooperation/behavior (*p* = 0.043), number of teeth requiring treatment (*p* = 0.018), and medical comorbidities (*p* < 0.001). Younger children (< 5 years) were more likely to undergo DGA due to extensive treatment needs, while older children (> 5 years) were more frequently referred due to behavioral issues and medical comorbidities (Table 5).

**Table 5:** Correlations between the child's age at the time of referral to DGA and DGA reasons (*N* = 234).

Documented reason for DGA		Age of the Child at referral for DGA *n* (%)		Total *n* (%)	*p.*
		<5 years *n* (%)	>5 years *n* (%)		
Cooperation and behavior	No	148 (75)	21 (58)	169 (72)	0.043
	Yes	50 (25)	15 (42)	65 (28)	
Number of teeth requiring treatment	No	79 (40)	22 (61)	101 (43)	0.018
	Yes	119 (60)	14 (39)	133 (57)	
Residential distance from the hospital	No	193 (98)	36 (100)	229 (98)	0.335
	Yes	5 (2)	–	5 (2)	
Health status	No	162 (82)	18 (50)	180 (77)	<.001
	Yes	36 (18)	18 (50)	54 (23)	
Parental preference	No	114 (58)	26 (72)	140 (60)	0.099
	Yes	84 (42)	10 (28)	94 (40)	

Pediatric dentists’ perspectives on DGA indications were gathered from 110 completed surveys. Respondent demographics are shown in Table 6, and their opinions on DGA indications are summarized in Table 7.

**Table 6:** Distribution of sociodemographic variables in the survey sample (*N* = 110).

Sociodemographic variables		*n*	%
Gender	Male	24	22
	Female	48	44
Primary place of work (more than one possible answer)	Private clinic	60	54
	Public clinic	60	54
	University	29	26
Do you regularly perform moderate sedation?	Yes	75	68
	No	35	32
Do you regularly perform DGA?	Yes	47	43
	No	63	57
Evaluation of the number of DGA/referrals performed by you in the last 10 years	Increased	49	45
	Unchanged	42	38
	Decreased	19	17

**Table 7:** Dentist perspectives on DGA indications.

Possible indication		*n*	%
Number of teeth needing treatment	4≥	16	15
	5≤	50	45
	Not relevant	44	40
Number of dental visits required (if not DGA)	4≥	45	41
	5≤	30	27
	Not relevant	35	32
Multiple extractions required	Yes	32	29
	No	52	47
	Not relevant	26	24
Multiple crowns required	Yes	48	44
	No	42	38
	Not relevant	20	18
Children with special needs	Yes	5	5
	No	105	95
Parents’ preference although the child is cooperative	Yes	18	16
	No	92	84
At least one treatment with sedation to evaluate behavior is mandatory before the referral decision	Yes	80	73
	No	30	27

Multivariate analysis revealed that dentists who regularly perform moderate sedation were less likely to consider multiple required dental visits (*p* = 0.042), multiple extractions (*p* = 0.028), multiple crowns (*p* = 0.047), or parental preference alone (*p* = 0.021) as indications for DGA compared to those who do not perform moderate sedation. Furthermore, dentists who regularly perform DGA were more likely to require a sedation visit to assess behavior prior to DGA referral (*p* = 0.003) and less likely to consider parental preference alone as an indication for DGA compared to dentists who do not perform DGA (*p* = 0.010).

## 4 Discussion

This study aimed to retrospectively map the reasons for referral to DGA, alongside the attitudes of pediatric dentists. The findings indicate that the most common reason for referral was extensive treatment needs, with an average of 11 teeth requiring treatment and a mean age at referral of 4 years. However, according to the practitioners’ attitudes, the extent of treatment alone was not a sufficient reason for referral. This example illustrates the complexity of the decision-making process regarding DGA referrals, which typically does not rely on a single reason but rather on a combination of factors.

The difficulty young children face with multiple dental visits, combined with the complexity of the dental treatment associated with non-cooperative behavior, often necessitates DGA (15). Age was the second most common reason for DGA. Studies indicate that cooperation improves with age (16, 17), whereas in younger, pre-cooperative children, pharmacological intervention is often required (18). Analysis of one-year DGA cases in a single private practice illustrates the association between young age and the extent of dental treatment as referral reasons. Campbell et al. (19) reported that the majority of cases were children younger than 6 years old, with an average number of teeth treated was approximately 9 in all age groups. Half of their participants had dental procedures on 10–18 teeth.

Parental preference was the third most common reason for DGA referrals. Notably, children whose parents expressed a preference for DGA exhibited higher levels of cooperation. This may be attributed to the parents’ own negative dental experiences or dental anxiety (20, 21). Although cooperation was relatively higher in this group, the mean Frankl scale score of 2.15 still indicates a low level of cooperation, suggesting negative behavior and justifying the parental desire for DGA (13). Alternatively, dentists might have documented ‘parental preference’ when parents opted for DGA despite the potential for alternative pharmacologic behavioral management. It is plausible that children with extremely low cooperation were recommended for DGA by the dentist, with ‘cooperation/behavior’ being the documented reason. Children treated under GA will achieve their treatment during a single session. Parents may opt for such treatment in comparison to prolonged and numerous treatment sessions and ignore concerns about the safety of each mode of treatment (21). With the increasing participation of parents in the decision process, the attitude of parents toward behavior management constitutes an important factor when a method of treatment is selected and parents need guidance in the decision-making process. The past decade has seen a revolution in public and professional attitudes toward the management of children (21). Djalali Talab and Geibel (22) compared parental and practitioners’ acceptance of DGA in pediatric patients. Both groups agreed that the extent of treatment and low compliance are a suitable indication for GA. Dentists were more likely to accept GA due to a mental disability than parents. Parents were more likely to accept GA than dentists when multiple extractions were needed (regardless of compliance) or when acute pain was present. Economic factors may also influence parental preference. In Israel, DGA is covered by national health insurance for children under 5 years old. Parents concerned about their child's cooperation during multiple appointments may prioritize utilizing this coverage. Conversely, some parents may prefer a single appointment, even at personal expense.

Lack of child cooperation/behavior was the fourth leading reason for DGA referrals. Notably, some children had only undergone dental examinations, while others had prior pharmacological behavioral management, including sedation. When sedation fails as a behavioral management technique, DGA becomes necessary. In this study, nearly half of the children receiving DGA had only a history of dental examinations. Interestingly, a significant number of dentists, particularly those who regularly perform DGA, preferred at least one attempt at sedation before DGA referral.

Medical comorbidities were the fifth most common reason for DGA referrals, documented in 23% of cases, despite 35% of the sample being medically compromised. This finding is undoubtedly influenced by the specific study population and might vary in a sample with a higher proportion of children with special health care needs. In some children with medical comorbidities, pharmacologic behavioral management other than DGA is contraindicated. DGA is also indicated in certain developmental disorders that impair behavior and cooperation (8). Medical comorbidities were more frequently documented as a referral reason in children older than 5 years. It is plausible that young age was the primary documented reason for DGA in medically compromised children under 5 years old. Parental preference was more often cited as a reason for DGA in healthy children. It is reasonable to infer that dentists would typically recommend alternative behavioral management for healthy children aged 5 and older. Therefore, parental preference, when documented as the reason for bypassing these methods, becomes particularly noteworthy.

Residential distance from the medical center was the least common reason for DGA referrals in our study, which contrasts with findings from other countries that have reported a correlation between residence and the need for GA (11). Given Israel's relatively small size, travel distance for consecutive dental visits may not be as significant as in larger countries. Moreover, Israeli health insurance funds are mandated to provide dental treatment solutions within 30 km of a patient's residence (23).

This study has several limitations. First, the reliance on existing medical records, resulted in incomplete data, such as missing residential information and Frankl scale scores for some patients. Frankl scores, which are crucial in evaluating the decision for DGA in children, were recorded in only 160 of the 245 patients. Furthermore, nearly one-quarter of potentially eligible cases (73/322) were excluded due to missing documentation of the reason for referral, which may potentially raise the possibility of bias. Additionally, the documentation of referral reasons was clinician-dependent, introducing subjectivity. Furthermore, parental preference was analyzed as a categorical variable, despite being derived from retrospective chart documentation rather than a standardized assessment tool. Future studies should develop and employ prospective, standardized methods to more accurately evaluate parental preferences. In addition, the study was conducted at a single medical center, limiting its generalizability to other centers or populations. Although data collection occurred during the COVID-19 pandemic, which may have influenced treatment decisions and preferences, only one lockdown occurred from January 8 to February 7, 2021. Notably, DGA services at the department remained operational during that time. Finally, the survey combined specialists and general practitioners into one group, despite probable variations in their clinical training and their access to specialized sedation or DGA services.In conclusion, the study results emphasize the complex clinical considerations that pediatric dentists assess before recommending general anesthesia. All factors must be evaluated together, rather than in isolation. The proposed decision-making algorithm begins with an assessment of the patient's health status. If the patient's medical condition precludes the use of non-pharmacological or moderate sedation techniques, DGA is indicated. For medically cleared patients, behavior is evaluated. Patients exhibiting positive cooperation are managed with behavior guidance or inhaled/moderate sedation, though clinicians must remain mindful of the patient's age and treatment extent, as these factors may lead to behavioral deterioration. For patients with negative cooperation, the clinician must weigh the suitability of pharmacological sedation against the comprehensive requirements of DGA. Partnership with parents and taking their preferences into account are also essential.

## Data Availability

The raw data supporting the conclusions of this article will be made available by the authors, without undue reservation.
